# Making the most out of the EPA Research Summer School: from a group exercise to an international collaborative study protocol

**DOI:** 10.1192/j.eurpsy.2022.1953

**Published:** 2022-09-01

**Authors:** C. Tapoi, R. De Filippis, C. Noël, D. Almeida, D. Gurrea Salas, K. Mieze, A. Pushko, M. Pinto Da Costa

**Affiliations:** 1 Prof. Dr. Alexandru Obregia Clinical Psychiatry Hospital, Psychiatry, Bucharest, Romania; 2 University Magna Graecia of Catanzaro, Department Of Health Sciences, Catanzaro, Italy; 3 Centre Hospitalier Universitaire Saint-Pierre , Department Of Child Psychiatry, Bruxelles, Belgium; 4 Hospital Prof. Doutor Fernando Fonseca, Department Of Psychiatry, Amadora, Portugal; 5 Psychiatric Services Aargau, Department Of Psychiatry And Psychotherapy, Brugg, Switzerland; 6 Riga Stradins University, Department Of Doctoral Studies, Riga, Latvia; 7 Ivano-Frankivsk National Medical University, Department Of Psychiatry,narcology And Medical Psychology, Ivano-Frankivsk , Ukraine; 8 King’s College London, Institute Of Psychiatry, Psychology & Neuroscience, London, United Kingdom

**Keywords:** Psychiatric trainee, EPA Summer School, Early career psychiatrist, training

## Abstract

**Introduction:**

The 2021 Research Summer School took place virtually, and 7 psychiatric trainees or early career psychiatrists (ECPs) from 7 different European countries participated in a working group on how to conduct a cross-sectional survey study.

**Objectives:**

To provide an overview of the process of developing an internationally collaborative protocol during the EPA Virtual Research Summer School.

**Methods:**

All participants were asked by the Faculty mentor chairing this working group to write a research question that could be investigated through a cross-sectional survey. After a brainstorming discussion, it was decided to investigate the experiences, knowledge, and attitudes of psychiatric trainees and ECPs about electroconvulsive therapy (ECT) in Europe, an effective yet controversial procedure.

**Results:**

The process of developing a protocol entailed different phases. First, a literature search was conducted, which supported the need to explore more the attitudes towards ECT among ECPs. Through group discussion the study’s objectives were decided, as well as the most appropriate methodology (including data collection and questionnaire use). At the end of the course, the core of the research plan was presented to all participants at the Research Summer School, preceding its implementation.

**Conclusions:**

Participating in the EPA Research Summer School is a unique experience, a great learning opportunity, and can also lead to fruitful collaborations. It enabled the learning of the key aspects of designing and conducting a survey. In a short period of time, it was possible to design a study protocol for a future international cross-sectional survey on ECT.

**Disclosure:**

No significant relationships.

